# Adaptation of Dubins Paths for UAV Ground Obstacle Avoidance When Using a Low Cost On-Board GNSS Sensor

**DOI:** 10.3390/s17102223

**Published:** 2017-09-28

**Authors:** Ramūnas Kikutis, Jonas Stankūnas, Darius Rudinskas, Tadas Masiulionis

**Affiliations:** Department of Avionics, Vilnius Gediminas Technical University, 02187 Vilnius, Lithuania; ramunas.kikutis@vgtu.lt (R.K.); jonas.stankunas@vgtu.lt (J.S.); darius.rudinskas@vgtu.lt (D.R.)

**Keywords:** UAV navigation, ground obstacle avoidance algorithm, Dubins paths, GNSS position measurements, position accuracy

## Abstract

Current research on Unmanned Aerial Vehicles (UAVs) shows a lot of interest in autonomous UAV navigation. This interest is mainly driven by the necessity to meet the rules and restrictions for small UAV flights that are issued by various international and national legal organizations. In order to lower these restrictions, new levels of automation and flight safety must be reached. In this paper, a new method for ground obstacle avoidance derived by using UAV navigation based on the Dubins paths algorithm is presented. The accuracy of the proposed method has been tested, and research results have been obtained by using Software-in-the-Loop (SITL) simulation and real UAV flights, with the measurements done with a low cost Global Navigation Satellite System (GNSS) sensor. All tests were carried out in a three-dimensional space, but the height accuracy was not assessed. The GNSS navigation data for the ground obstacle avoidance algorithm is evaluated statistically.

## 1. Introduction

Nowadays, small unmanned aerial vehicles (UAVs) are becoming increasingly popular and are being more widely used for various practical applications. These vehicles can be used for various missions, such as small cargo delivery (e.g., delivering an automated external defibrillator) [1], electrical line monitoring or visual inspection of other ground infrastructure with a difficult access [2], fast response for post-earthquake consequences and initial help [3], surveillance, mapping and 3D visualization of ground objects [4], as a tool to get photos and other information for landscape and urban planning [5], etc. In spite of the wide application, a lot of restrictions at the international and national levels are issued by such organizations as the European Aviation Safety Agency (EASA), the Federal Aviation Administration (FAA) or local Civil Aviation Administrations (CAAs) [6]. Since these restrictions for UAV flights are due to the current lack of flight safety and reliability, research in various UAV related fields is of great importance. Researchers are searching for methods to ensure that automated UAV flights meet national airspace restrictions and limitations [7]. Otherwise, it will be impossible to implement and use small UAVs for all practical applications.

Research on the flight control systems of UAVs has a direct relationship with flight navigation accuracy and flight safety. This field is usually subdivided into three branches: guidance, navigation, and control. These areas can be analyzed at once, as in the paper on the control systems of two cubic blimps [8], or can be separated and analyzed independently. While research in these fields is important for UAV flight safety, currently, the greatest focus is on new navigation systems and sensing technologies, such as visual odometry, target relative navigation, terrain relative navigation, simultaneous localization and mapping, simultaneous mapping and planning, and safe landing area detection. Guidance systems are also widely analyzed. Techniques from navigation systems and sensing technologies are applied for various flight procedures, whereas algorithms are used for mission planning, path planning and multi-UAV cooperation. Research in this field includes studies on potential fields, when a UAV mission is generally controlled by using the potential field to steer the UAV in the correct direction [9,10], and optimization methods, such as finding the most efficient flight trajectory, for example, in an urban environment [11]. Other studies focus on planning navigation under uncertainties, coordinated flight control, cooperative perception or coordinated flights with path planning by avoiding ground obstacles [12].

One of the goals for UAV guidance systems emphasized in recent research is path planning under uncertainties, which can be achieved in different ways. Some papers analyze the use of camera sensors to detect ground obstacles, while others propose combinations of various low cost sensors [13]. If a camera is used, the methods for obstacle detection, avoidance and UAV control can be implemented in areas even without a Global Navigation Satellite System (GNSS) signal [14]. Camera images can be used for ground obstacle detection and identification, tracking of other UAVs, and, if needed, as a navigation data sensor for other ground obstacle avoidance algorithms [15]. The image depth may be used to predict the distance to the obstacle [16] and, in some cases, can be shared between several unmanned vehicles [17]. Using these camera-based information methods, even auto-landings can be implemented [18]. However, in any case, the camera placement position and all angles should be calculated [19].

Obstacle detection is only one of the goals; others include the accurate avoidance of a ground obstacle or flights in a complex environment [20] and require the implementation of different methods or algorithms to attain them. If, for example, the ground obstacles are of various sizes and shapes or have a different height, different mapping techniques should be applied, as demonstrated in [21] and, where ellipsoidal geometry is used, in [22]. However, sometimes it is necessary to generate additional waypoints to safely overfly or to fly around a ground obstacle [23], or to change some additional UAV flight parameters (e.g., roll angle, airspeed) or the controller [24].

As noted earlier, if we want to use small UAVs for various practical applications, we need both to safely overfly or fly around the ground obstacles, and to meet all legal restrictions on UAV flights. One of the methods to attain this goal is to use Dubins paths. In the next section, a general idea of Dubins paths and their methodology is provided together with a review on current research on UAV navigation and obstacle avoidance.

## 2. Dubins Paths Methodology

In geometry, the term Dubins path typically refers to the shortest curve that connects two points. The calculations can be done in either a two-dimensional or a three-dimensional space with a constraint on the path’s curvature and with prescribed initial and terminal tangents to the path. More precisely, for Dubins paths, each navigation leg corresponds to either the form CSC or CCC, where C stands for a concave or convex circle segment, and S—for a line segment. These flight path solutions are commonly called Dubins paths [25]. A general idea of Dubins paths and their implementation is proposed in [26,27]. Moreover, if we need to use in our research flight dynamics and kinematics, Dubins paths problems can be approached by using the Dubins aircraft model, which is presented and used in [28]. In some of the studies, Dubins paths are used in the Dubins traveling salesman problem (DTSP), which refers to a Dubins vehicle. This vehicle moves at a constant speed, cannot move in reverse, and has a minimum turning radius. Dubins vehicles are widely used for the motion planning of aircraft, ships or UAVs [25].

**Figure 1:**
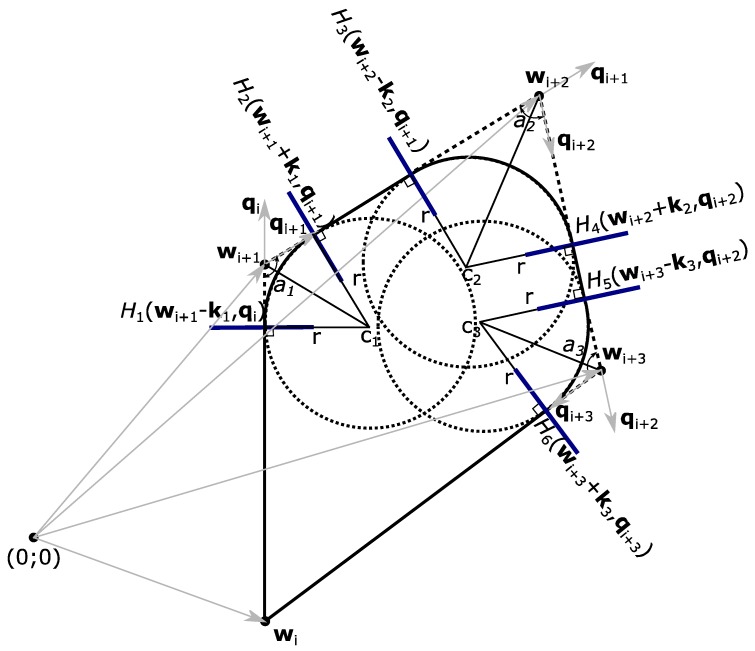
Conversion of a simple UAV mission into a Dubins trajectory.

For UAVs, a simple flight mission, which has some straight flight segments, can be converted into Dubins paths by inserting filleted circular arcs near the turning points, as shown in Figure 1, which illustrates smoothed flight trajectory by inserting fillets near the turning points thus forming Dubins paths.

To get the numerical data for the transition from the first flight segment which is connected by two navigation points **w**_i_ and **w**_i+1_ to the flight segment with points **w**_i+1_ and **w**_i+2_, unit vectors $q_{i}$ and $q_{i+1}$ (which show the directions of both flight segments) are needed. Unit vectors $q_{i}$ and $q_{i+1}$ can be calculated using Equations (1) and (2):

(1) $$q_{i}=\frac{w_{i+1}-w_{i}}{\left\|w_{i+1}-w_{i}\right\|}$$

(2) $$q_{i+1}=\frac{w_{i+2}-w_{i+1}}{\left\|w_{i+2}-w_{i+1}\right\|}$$

Since a UAV needs a command to switch from a straight to a circular flight path segment, the coordinate where the transition happens has to be defined. For this reason, angles *a* are needed, which are found by using Equation (3):

(3) $$∠a_{x}=\cos^{-1}(-q_{i}^{T}q_{i+1})$$

Then, using angle *a* and the UAV turning radius, the distance from waypoint **w**_i+1_ for the transition between flight segments is found. With unit vectors **q** and distances **k** as in Equation (4), planes *H*_1_ and *H*_2_ can be found, representing the coordinates where a UAV does the transition from a straight flight segment into a turning—circular flight segment and then switches from a circular into a straight one by using Equations (5) and (6):

(4) $$k_{1}=-\left(\frac{r}{\tan\frac{∠a_{1}}{2}}\right)q_{i+1}$$

(5) $$H_{1}\left(w_{i+1}-k_{1},q_{i}\right)$$

(6) $$H_{2}\left(w_{i+1}+k_{1},q_{i+1}\right)$$

If a UAV crosses the coordinates of plane *H*_1_ or *H*_2_, the transition is made. For complete calculations, the flight coordinates of the circular arcs must also be known. In this case, the UAV needs to know the center points of the Dubins circle which can be calculated with Equation (7):

(7) $$c_{1}=w_{i+1}-\left(\frac{r}{\sin\frac{∠a_{1}}{2}}\right)\frac{q_{i}-q_{i+1}}{\left\|q_{i}-q_{i+1}\right\|}$$

The same technique can be used for all waypoints.

This or an equivalent methodology is used in various research papers. For example, a Dubins paths navigation methodology was proposed to achieve more precise and accurate UAV flights in [26,27,29,30]. In these papers, one of the tasks is to use the Dubins paths. For example, in [30], the authors propose to solve the Generalized Dubins Path Problem (GDPP), whereas in [27], a mathematical derivation of the Dubins paths is presented. In [27], the authors also use UAV flight dynamics and apply a vector field to keep the UAV on the generated Dubins paths flight mission. However, a great part of these papers is concerned with adapting Dubins paths to generate a path with the shortest distance or to search for the shortest time distance [10,31,32,33,34]. For example, as stated in [10], the UAV joins a predesigned admissible circular trajectory around a target, which is a fixed point space. Afterwards, this method is used to generate a time-optimal control synthesis for tracking a circle by a Dubins vehicle [10]. In other studies, Dubins paths and other autonomous UAV navigation techniques are also evaluated in windy conditions [35]. In [31], the authors consider the problem of determining a time-optimal path for a fixed-wing Miniature Air Vehicle (MAV) also taking into account the wind. As an alternative to wind, in [34], the authors try to find the minimum time of Dubins paths in the presence of a mathematically generated drift field.

In some publications on Dubins paths, optimization methods are applied or updated for ground obstacle avoidance, as proposed in [36]. In this paper, Dubins paths were applied for both static and moving ground obstacle avoidance by using a variation of the Rapidly-exploding Random Tree (RRT) planner. Very similar research was done by [37]. In this study, a combination of both static and moving obstacles was used in an enclosed territory, as well as a quadcopter and a developed search-and-avoid algorithm for obstacles. Search-and-avoid algorithms for Dubins paths can be developed by using several different techniques that fall broadly into three categories:

1. Methods where a suitable order of the obstacles is determined first and then suitable overflight directions are found;
2. Methods where suitable overflight directions are found first and then the order of the obstacles is optimized;
3. Methods that do not separate the order of operations (an overflight direction is found and an order of obstacles is chosen at the same time).

These algorithms when Dubins paths are used can also be applied to avoid moving aerial obstacles—other UAVs or aircraft [38].

Various ground obstacle avoidance methods are proposed in different studies. For example, in [39], a virtual force field is generated around the ground obstacle. This field has various strength levels, depending on how close to the obstacle the vehicle is. Based on the field strength, a vehicle makes either smaller or larger corrections to avoid a collision. In [40], the authors generate an obstacle avoidance algorithm by adding three additional flight legs around the ground object. Still, these flight legs cannot represent real Dubins paths.

Obstacle avoidance in an open territory is an even harder task. In [41], convex polygon flight paths, which should be used to avoid ground obstacles of various shapes, are generated. Alternatively, as in [42], a certain safety buffer zone around the ground obstacles is applied and Dubins paths are used to look for an optimal flight path.

A great part of the related papers tackle ground obstacle avoidance problems with quadrocopters. In most cases, these problems are related to either obstacle detection or the attempt to find an optimal flight path. However, in most of these papers, there is a notable gap, as the studies do not evaluate the safety area around the obstacle. Taking into consideration the regulations on UAV flights, the accuracy of the flight path to avoid a ground obstacle should be evaluated as well. Therefore, in this study, Dubins paths are applied for safe UAV ground obstacle avoidance while complying with UAV regulations. We propose to generate additional waypoints, assuming that the ground obstacle coordinates are known (could be found by using camera sensing or other discussed methods) and are given to a UAV. One of the methods to test UAV navigation performance is to use some simulation technique like “MAV3DSim” [43]. In our case the research is done by using Software-in-the-Loop (SITL) simulation and a low cost GNSS sensor, which was attached to a flying-wing UAV. A new UAV navigation and guidance algorithm is derived by using GNSS sensor coordinates and simulated ground obstacle coordinates. In the next section, the standard methodology of Dubins paths for UAV flights is applied to show the mathematics of our obstacle avoidance algorithm.

## 3. Research Methods for Ground Obstacle Avoidance

In this section, the derivation of a mathematical model for an autonomous UAV flight which can be used to safely avoid ground obstacles is introduced.

**Figure 2:**
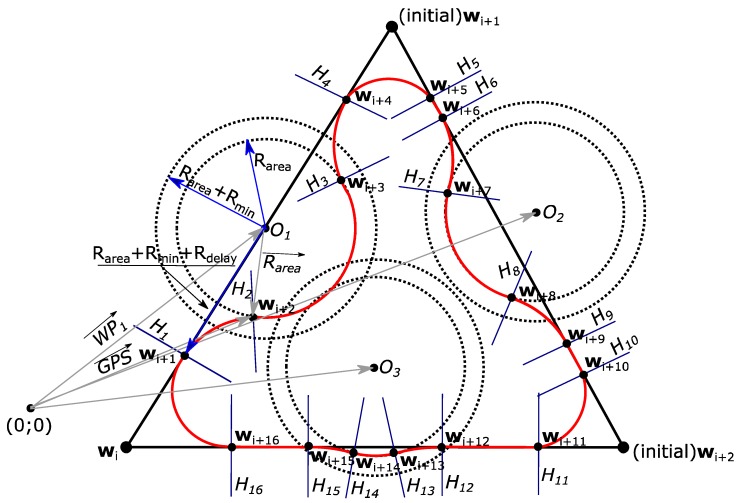
Flight mission with three ground obstacles that should be automatically avoided.

It is assumed that a triangular shaped trajectory, which starts at waypoint **w**_i_, is to be flown by using Dubins paths near the turns. Also, all ground obstacles need to be automatically avoided by a safe distance. This scenario is represented in Figure 2.

In Figure 2, the coordinates of the ground obstacles are represented by *O*_1_, *O*_2_ and *O*_3_. In order to safely fly around the ground obstacles, at least a radius R_area_ must be flown, which is the minimum safe distance defined by national or international legal organizations. However, during the turn, the UAV will need some additional distance to get to the boundary of the minimum safe distance from the obstacle. In this case, the distance from the obstacle must be increased to the distance R_area_ + R_min_, where R_min_ can be found from Equation (8):

(8) $$R_{\min}=\frac{V^{2}}{g\times {\mathrm{tg}\theta }_{\max}}$$

The R_min_ depends on true air speed, V, and the maximum roll angle, θ_max_. Finally, the UAV will experience a certain time lag before starting the turn, so the final distance from the obstacle where the turn action must be initiated is **R**_area_ + **R**_min_ + **R**_delay_. The approximate delay of the UAV decision is found by Equation (9), but can be adjusted for tuning purposes:

(9) $$R_{\mathrm{delay}}=2.5\cdot V$$

To fly around the ground obstacle safely, four additional waypoints are generated, and their exact coordinates are needed. At these points, the planes from *H*_1_ to *H*_16_ must be located to indicate a UAVs transition from one flight segment to another flight segment. As the current position of the UAV is measured by using a GNSS sensor, with the ground obstacle location at a constant value, the coordinate at which one of the *H* planes must be located is found by using Equation (10):

(10) $$GPS=O_{i}-R$$

In Equation (10), vector **R** is expressed by using Equations (11) and (12).

(11) $$R=R_{\mathrm{area}}+R_{\min}+R_{\mathrm{delay}}$$

(12) $$R_{\mathrm{area}}=R_{1}+R_{\mathrm{err}}$$

where **R**_err_ is the radius calculation error, as in Equations (13) and (14). In this case, the **R**_err_ value inside of the length **R**_1_ is found initially, and this geometrical calculation uncertainty is compensated later by adding **R**_err_ to **R**_1_, as shown in Equation (12):

(13) $$R_{\mathrm{err}}=R_{1}-\sqrt{R_{1}^{2}-{\left(\frac{k}{2}\right)}^{2}}$$

(14) $$R_{\mathrm{err}}=R_{1}-\sqrt{R_{1}^{2}-{\left(\frac{\frac{V^{2}}{\cos\left(\mathrm{tg}\left(\frac{\left|GPS_{x}-O_{x}\right|}{\left|GPS_{y}-O_{y}\right|}\right)\right)g\cdot \mathrm{tg}\theta_{\max}}}{2}\right)}^{2}}$$

**Figure 3:**
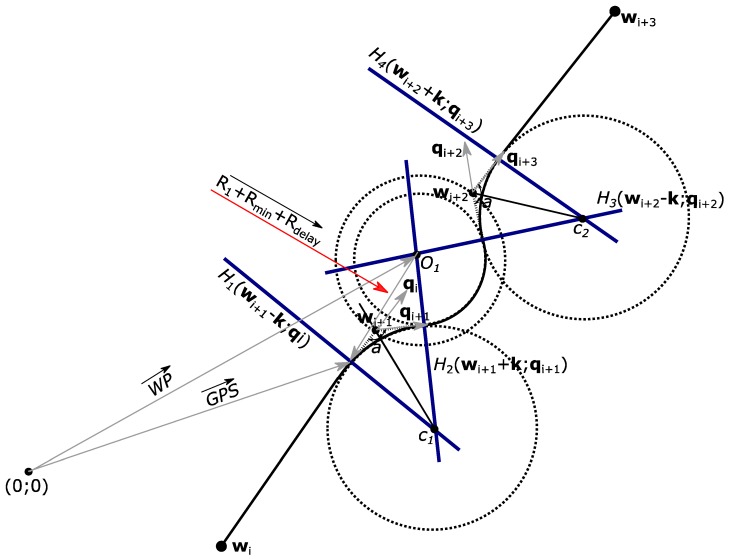
Additional waypoint and planes for one flight mission segment to safely fly around ground obstacle O_1_.

A more detailed analysis of the application of Dubins paths for one general straight path flight segment and one ground obstacle is illustrated in Figure 3.

As shown in Figure 3, plane *H*_1_ is not exactly on the position of waypoint **w**_i+1_ which is found from Equation (3), but is in the same direction where the unit vector **q**_i_ points to by distance **k** from this plane. According to Dubins paths theory, Distance **k** can be found by using Equation (15):

(15) $$k=\left(\frac{r}{\tan\frac{∠a}{2}}\right)q_{i}$$

**Figure 4:**
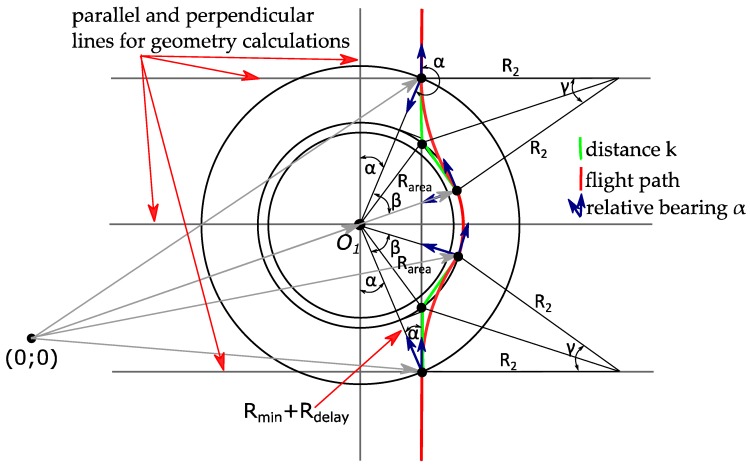
Geometry of ground obstacle avoidance.

In Equation (6), however, *r*—the radius of the Dubins circle with the center C_1_ and ∠*a,* the size of which depends on how much the UAV changes its course—is still unknown. For this reason, flight, trajectory geometrical calculations, which are visualized in Figure 4, are applied.

To begin with, an equation is used to find the unit vector **q**_i_, which shows the flight path direction from the initial waypoint to the next waypoint, without paying attention to ground obstacles. According to Dubins trajectory theory, vectors **q**_i_ can be found from Equation (16):

(16) $$q_{i}=\frac{w_{i+3}-w_{i}}{\left\|w_{i+3}-w_{i}\right\|}$$

As shown in Figure 4, distance *k* can be found by using Equation (17), and the UAV relative bearing angle can be calculated by using the live GNSS on-board sensor data for Equation (18).

(17) $$k\approx \frac{R_{\min}{+R}_{\mathrm{delay}}}{\cos\left(\alpha \right)}$$

(18) $$∠\alpha =\mathrm{tg}\left(\frac{\left|GNSS_{x}-O_{x}\right|}{\left|GNSS_{y}-O_{y}\right|}\right)$$

By inserting Equations (8) and (18) into Equation (17), the calculation of distance *k* based on the known parameters is obtained, as in Equation (19):

(19) $$k\approx \frac{1}{\cos\left(\mathrm{tg}\left(\frac{\left|GNSS_{x}-O_{x}\right|}{\left|GNSS_{y}-O_{y}\right|}\right)\right)}\times \left(\frac{V^{2}}{g\times {\mathrm{tg}\theta }_{\max}}+R_{\mathrm{delay}}\right)$$

Distance *k* should only be calculated when the coordinate values received from the GNSS sensor meet the requirements for value **R**, found from Equations (10)–(12). This condition can be expressed by Equation (20):

(20) $$WP-\mathrm{GNSS}\leq R_{1}+R_{\mathrm{err}}+R_{\min}+R_{\mathrm{delay}}$$

When the condition in Equation (20) is met, distance *k* is calculated from Equation (19) and is converted into a vector form by using either Equation (21) or Equation (22):

(21) $$k=k\cdot q_{i}$$

(22) $$k=k\cdot \left(\frac{w_{i+3}-w_{i}}{\left\|w_{i+3}-w_{i}\right\|}\right)$$

By inserting *k* into Equation (22), a representation of vector **k** with the known values is obtained, as shown in Equation (23):

(23) $$k\approx \left(\frac{1}{\cos\left(\mathrm{tg}\left(\frac{\left|GNSS_{x}-O_{x}\right|}{\left|GNSS_{y}-O_{y}\right|}\right)\right)}\times \left(\frac{V^{2}}{g\times {\mathrm{tg}\theta }_{\max}}+2.5\times V\right)\right)\left(\frac{w_{i+3}-w_{i}}{\left\|w_{i+3}-w_{i}\right\|}\right)$$

Then, the position of plane *H*_1_ is represented by Equation (24).

(24) $$H_{1}\left(w_{i+1}-k,q_{i}\right)$$

To find the coordinates of plane *H*_2_, the unknown angles β, γ and the Dubins circle radius R_2_ are necessary. From the flight path geometry expressed in Figure 4, we derive the magnitude of angle β. Its value can be calculated either with Equation (25) or, if all known values are inserted—with Equation (26):

(25) $$∠\beta =\frac{l\cdot 180}{\pi R_{1}}+l\sin\alpha$$

(26) $$∠\beta =\frac{\frac{V^{2}}{g\times {\mathrm{tg}\theta }_{\max}}\left({180\;+\;\pi R}_{1}\right){\mathrm{tg}}^{2}\left(\frac{\left|GNSS_{x}-O_{x}\right|}{\left|GNSS_{y}-O_{y}\right|}\right)}{{\pi R}_{1}}$$

As is known form flight geometry, the waypoint relative bearing from an airplane’s position during the turn must change from the initial value, angle α, to a new value of 90°, and angle γ can be calculated by either using Equation (27) or Equation (28) if all known values are used:

(27) ∠γ=90°−α−β

(28) $$∠\gamma =90-\mathrm{tg}\left(\frac{\left|GNSS_{x}-O_{x}\right|}{\left|GNSS_{y}-O_{y}\right|}\right)-\frac{\frac{V^{2}}{g\times {\mathrm{tg}\theta }_{\max}}\left({180\;+\;\pi R}_{1}\right){\mathrm{tg}}^{2}\left(\frac{\left|GNSS_{x}-O_{x}\right|}{\left|GNSS_{y}-O_{y}\right|}\right)}{{\pi R}_{1}}$$

Then, R_2_ is obtained from Equation (29) or Equation (30):

(29) $$R_{2}=k\cdot \mathrm{tg}\frac{\gamma }{2}$$

(30) $$\begin{array}{l} R_{2}=\left(\frac{1}{\cos\left(\mathrm{tg}\left(\frac{\left|GNSS_{x}-O_{x}\right|}{\left|GNSS_{y}-O_{y}\right|}\right)\right)}\times \left(\frac{V^{2}}{g\times {\mathrm{tg}\theta }_{\max}}{+R}_{\mathrm{delay}}\right)\right)\cdot \\ \mathrm{tg}\left(45-\frac{1}{2}\cdot \mathrm{tg}\left(\frac{\left|GNSS_{x}-O_{x}\right|}{\left|GNSS_{y}-O_{y}\right|}\right)-\frac{\frac{V^{2}}{g\times {\mathrm{tg}\theta }_{\max}}\left({180\;+\;\pi R}_{1}\right){\mathrm{tg}}^{2}\left(\frac{\left|GNSS_{x}-O_{x}\right|}{\left|GNSS_{y}-O_{y}\right|}\right)}{{2\pi R}_{1}}\right) \end{array}$$

When the radius R_2_ is found, we look for angle *a* using Equation (31). Then, this value must be used for a UAV to find the Dubins circle center coordinate and the unit vector **q**_i+1_:

(31) $$∠a=\mathrm{arctg}\frac{R_{2}}{k}$$

The unit vector **q**_i+1_ is found as the vector’s **q**_i_ rotation as in Equation (32):

(32) $$q_{i}=\left[\begin{array}{cc} \cos(180-a) & -\sin(180-a)\\ \sin(180-a) & \cos(180-a) \end{array}\right]q_{i+1}$$

Having vectors **q**_i_, **q**_i+1_ and distance *k*, we find the Dubins circle center coordinates from Equation (33), and the position of the next plane, *H*_2_—from Equation (34):

(33) $$c_{1}=w_{i+1}-\left(\frac{R_{2}}{\sin\frac{∠a}{2}}\right)\frac{q_{i}-q_{i+1}}{\left\|q_{i}-q_{i+1}\right\|}$$

(34) $$H_{2}\left(w_{i+1}+k,q_{i+1}\right)$$

Plane *H*_2_, which is expressed by Equation (34), provides the second point where the UAV makes the transition from flying along the circular arc of one Dubins path to another circular arc, which is used to fly around the obstacle. The two remaining plane positions, *H*_3_ and *H*_4,_ and the additional waypoint **w**_i+2_ would be symmetrical to the previous *H*_1_, *H*_2_ and **w**_i+1_. In this case, the procedure for finding these values is identical and would be repeated for other obstacles.

As the derivation of obstacle avoidance model is an important part, UAV also needs to know the rules how and when to perform these calculations. For this reason, in next section we introduce exact algorithm to avoid ground obstacles.

## 4. Ground Obstacle Avoidance Algorithm

**Figure 5:**
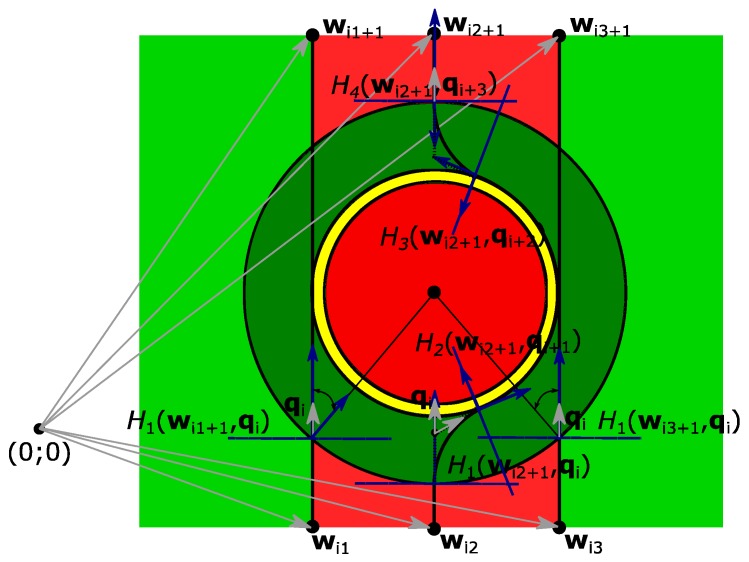
Decision areas for UAV ground obstacle avoidance algorithm: dark red—prohibited to fly area; yellow—additional safety area due to the calculations errors; dark green—area for the UAV to make an initial turn for the ground obstacle avoidance; light red—UAV approaching from there will need to make an obstacle avoidance; light green—UAV approaching from there will not need to make an obstacle avoidance.

In order to avoid ground obstacles, UAVs must make correct decisions. It is not enough to find only the required variables and unknowns. For this case, special sectors around the obstacle are defined, as represented in Figure 5.

As known from previous equations, the red, yellow, and dark green areas represent special areas around the obstacle: ground obstacle safety area, calculations error area and UAV turn area, respectively. There are also two additional areas: light green and light red. If a UAV is approaching an obstacle from the light red area, it will need to make an action to fly around the obstacle. However, if a UAV is arriving from the light green area, no additional action will be needed, and it will continue straight forward.

As shown in Figure 5, if a UAV is flying from waypoint **w**_i2_ to waypoint **w**_i2+1_, it arrives first at a coordinate of plane *H*_1_(**w**_i2+1_,**q**_i_*)*, which is located within distance **R** from ground obstacle O_1_, as in Equation (11). An on-board GNSS sensor determines the UAV’s position, but the obstacle’s coordinates should be known and kept in the UAV’s memory. When the UAV is at a position located in plane *H*_1_(**w**_i2+1_,**q**_i_*)* (the distance from the ground obstacle is **R**), it has not determined if it is safe to continue in this direction without any change of course. Therefore, the following action for the UAV is to evaluate the relative bearing from the ground obstacle, which is calculated by Equation (35):

(35) $$∠\mathrm{RB}=180-\cos^{-1}(-q_{i}^{T}q_{O_{i}})$$

Then, it must be checked if the |*RB|* is below the maximum RB_max_ for ground obstacle avoidance. RB_max_ can be found using Equation (36):

(36) $$∠{\mathrm{RB}}_{\max}=\sin\left(\frac{R_{\mathrm{area}}}{R}\right)$$

**Figure 6:**
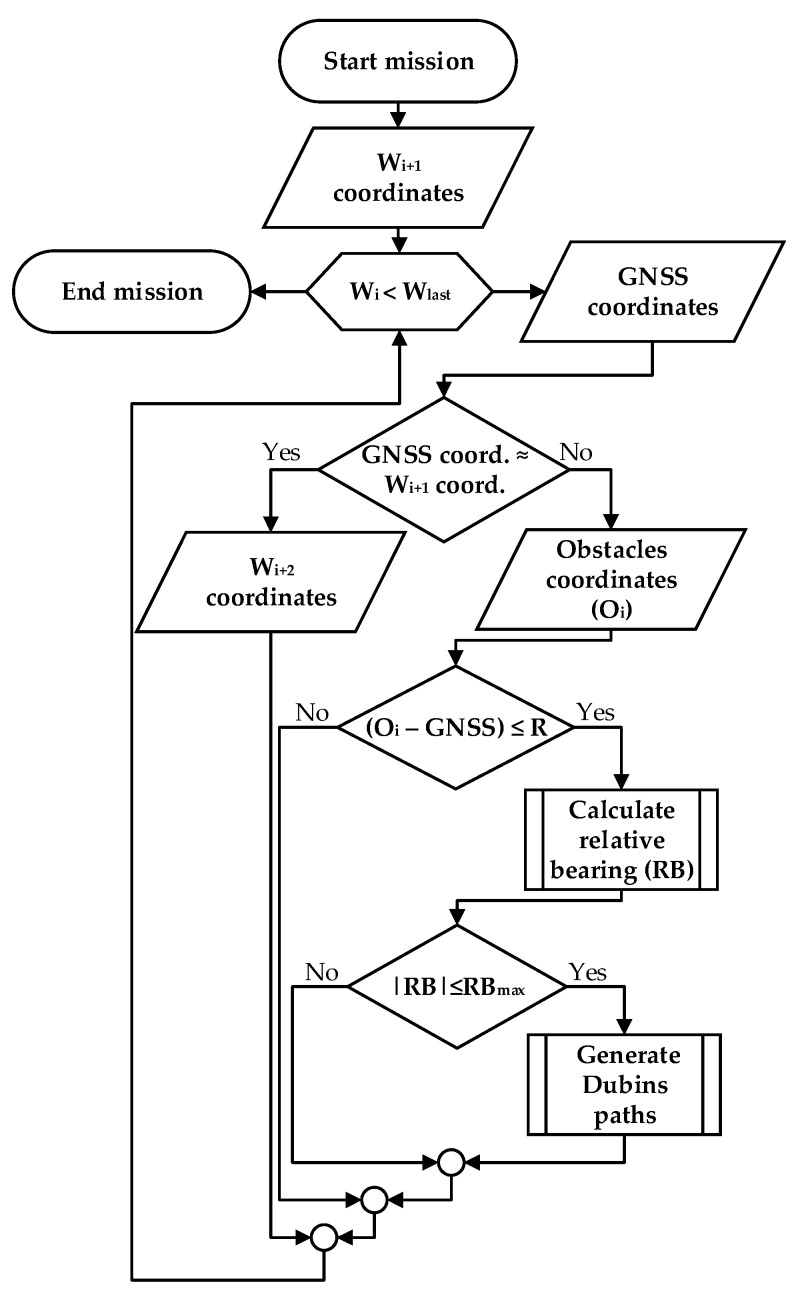
Algorithm for UAV ground obstacle avoidance.

If |RB| < RB_max_, the UAV must generate several Dubins path circles around the ground obstacle. These Dubins paths would be located between planes *H*_1_(**w**_i2+1_,**q**_i_*)* and *H*_2_(**w**_i2+1_,**q**_i+1_*)*, *H*_1_(**w**_i2+1_,**q**_i+1_*)* and *H*_2_(**w**_i2+1_,**q**_i+2_*)*, and *H*_1_(**w**_i2+1_,**q**_i+2_*)* and *H*_2_(**w**_i2+1_,**q**_i+3_*)*. The described flight procedure is illustrated in Figure 6 as a UAV actions algorithm.

This procedure is repeated until the UAV completes the entire flight mission. If there were no ground obstacles on the path, the UAV would omit this ground obstacle avoidance procedure and continue flying a regular flight mission. In the next section, the hardware and software used to test the new algorithm are presented.

## 5. Experimental Approach

Two methods have been chosen to test the algorithm: Software-in-the-Loop simulation (SITL) and real flight experiments. The Software-in-the-Loop simulation was done by using the ArduPilot autopilot software, whereas real flights were done with a more advanced autopilot—the Pixhawk 2 (PX4). Both tests were carried out by taking coordinate measurements with either a real or a simulated GNSS receiver, the “Ublox Neo-M8N”. The main technical characteristics of used GNSS receiver are presented in Table 1.

As we can see from the Table 2, it is stated that “Ublox Neo-M8N” has horizontal position accuracy of about 2.0–2.5 m. However, this accuracy is only valid if we make data logging in a static position for about 24 h. During UAV flight GNSS receiver accuracy would be much worse as we need to make real time measurements in a dynamic mode. In order to check accuracy of the new algorithm and GNSS receiver influence on that algorithm, we start with SITL simulation.

**Figure 7:**
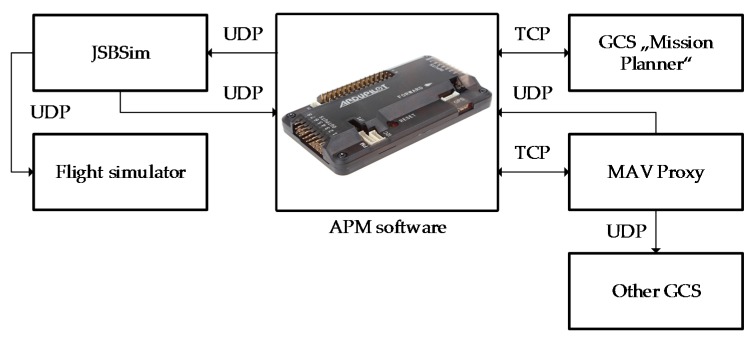
Software-in-the-Loop simulation setup.

The SITL configuration is shown in Figure 7.

For simulation purposes, an open source ArduPilot “Mega” (APM) autopilot software was used. The flights were set up by using two ground stations: the Ground Control Station (GCS), referred to as “Mission planner 1.3.44”, and the MAV Proxy on the APM console. The Transmission Control Protocol (TCP) and the User Datagram Protocol (UDP) were used as communication protocols. This simulation setup allowed using additional flight simulators to expand visualization, but in our case that was not necessary. In this case, the GNSS data were simulated by using the software and data communication protocols described earlier.

**Figure 8:**
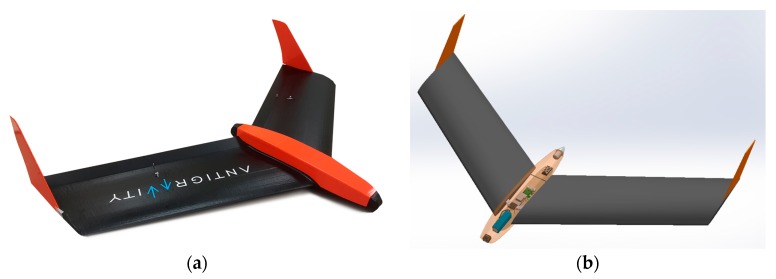
Research UAV: (**a**) Manufactured UAV; (**b**) UAV prototype in “Solidworks”.

Further real flight experiments were performed with a flying-wing UAV which was designed with “Solidworks”. It has a mass of 1.8 kg and a wingspan of 1.6 m. Its maximum flight time is 1 h and 35 min with a maximum flight distance of approximately 30 km. The UAV was equipped with the Pixhawk 2 autopilot, which was used to simulate ground obstacles and to test the new algorithm. UAV model is shown in Figure 8a and its “Soliworks” model is represented in Figure 8b. 

**Figure 9:**
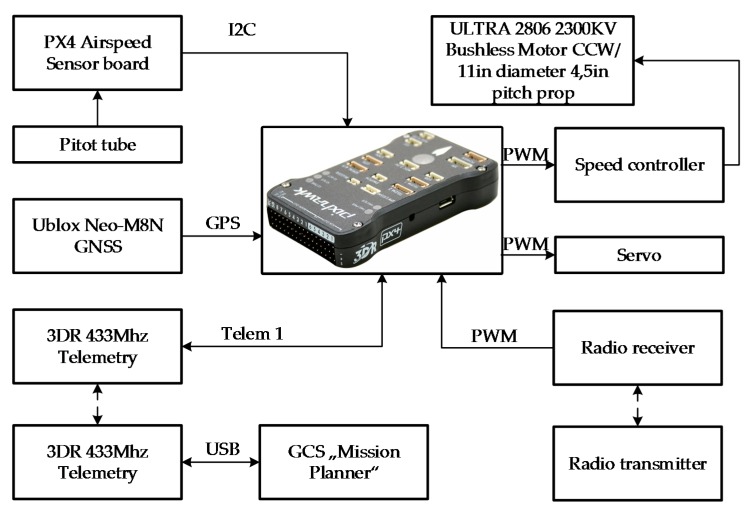
Setup of the aircraft equipment for the experiment.

The complete setup of the UAV with all its hardware inside the fuselage is shown in Figure 9. The main flight data were collected by using a “Pixhawk 2” autopilot and were recorded into the internal memory for later analysis. Live data were checked by using 433 MHz telemetry units and was sent to ground station “QGroundControl v3.1.3”. The most important technical specification of the “Pixhawk 2” autopilot are presented in Table 3.

The Software-in-the-Loop and real flight experiments were set up for a UAV to fly an equilateral triangle with a side length of 500 m (the same mission as in Figure 2). Three imaginable ground obstacles were added to this trajectory. With this setup, after all flights, experiment data were recalculated into the East-North-Up (ENU) coordinate frame by an inversion matrix, as in Equation (37):

(37) $$\left[\begin{array}{c} E\\ N\\ U \end{array}\right]=\left[\begin{array}{ccc} -\sin\lambda_{r} & \cos\lambda_{r} & 0\\ -\sin\phi_{r}\cos\lambda_{r} & -\sin\phi_{r}\sin\lambda_{r} & \cos\phi_{r}\\ \cos\phi_{r}\cos\lambda_{r} & \cos\phi_{r}\sin\lambda_{r} & \sin\phi_{r} \end{array}\right]\left[\begin{array}{c} \Delta LON\\ \Delta LAT\\ \Delta ALT \end{array}\right]$$

In Equation (37), λ_r_ indicates the reference longitude and ϕ_r_—the reference latitude. These values are used as the main coordinates at which the conversion to ENU is done. Δ*LON*, Δ*LAT*, and Δ*ALT* are the difference between the UAV’s position and a reference coordinate. The ENU coordinates of the flight mission and ground obstacles are shown in Table 4. Additional waypoints are calculated by the UAV during the mission.

For experimental purposes, a safe radius of 50 m around the obstacle was chosen and remained constant during the experiments. In the next section, we present the results of the simulation and real flights as well as the comparison of the data.

## 6. Results

**Figure 10:**
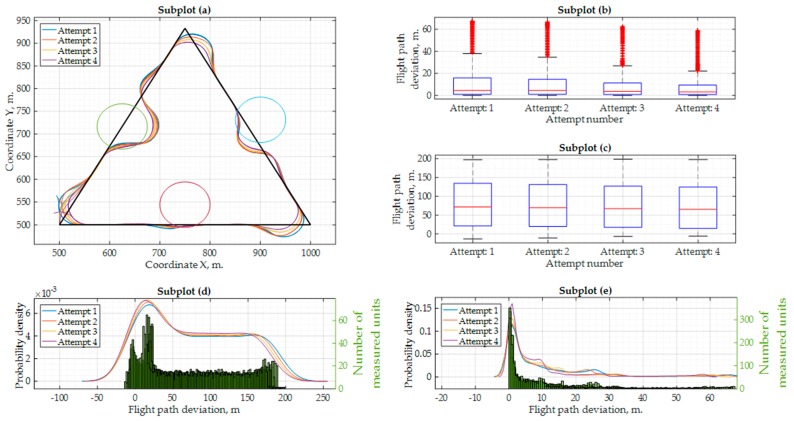
Results of the Software-in-the-Loop simulation with the “ArduPilot” autopilot with the flight speed at 15 m/s: (**a**) flight mission, flight trajectory and ground obstacles; (**b**) flight deviation from the standard flight mission path (when using ground obstacle avoidance); (**c**) flight deviation from the ground obstacle area boundary of 50 m. (when using ground obstacle avoidance); (**d**) probability density values and quantity of measurements for every flight path deviation value (standard mission); (**e**) probability density values and quantity of measurements for every flight path deviation value (from ground obstacle area boundary).

Firstly, the Software-in-the-Loop experiment was carried out. The autopilot was setup to fly at 15 m/s. For this experiment, four attempts with three laps were done for the flight mission illustrated in Figure 2. It was realized that the “ArduPilot” has some delay in decision-making—it tends to undershoot or overshoot the trajectory during the turn. The trajectories with undershoots and overshoots are visible in Figure 10a. In this figure, the circles represent the 50 m radius area around the ground obstacle positions (the minimum safety area around an obstacle, as stated in various UAV flight regulations). Each attempt was done with a different UAV delay configuration. As the results illustrated in Figure 10 show, the UAV was not able to fly around these obstacles safely. Figure 10b illustrates the statistics of the trajectory deviation from the triangular flight path with ground obstacles. In Figure 10c, the same statistics is provided, but the trajectory deviation is only measured from the 50 m ground obstacle boundary area. To avoid negative values, which represent that a UAV is inside of the 50 m ground obstacle boundary area, some corrections were made by tuning the UAV delay. As seen in Figure 10c, during attempt 4, the lowest quartile was closer to 0. It means that for this attempt 25% of the flight data (coordinates) had a slightly greater accuracy. Although, as seen in Figure 10d, the probability density is the highest for a trajectory deviation of 15–20 m (from the 50 m obstacle boundary) and is approximately 6,7·10^−3^–7,2·10^−3^. Furthermore, the probability density of being exactly where we need to be (0 m deviation) is approximately 5,3·10^−3^–6,0·10^−3^. If only the flight trajectory is considered, the flight path deviation is smaller and has the greatest probability density that a UAV will be on the path of 0.15. Other important statistical data for this experiment is shown in Table 5.

**Figure 11:**
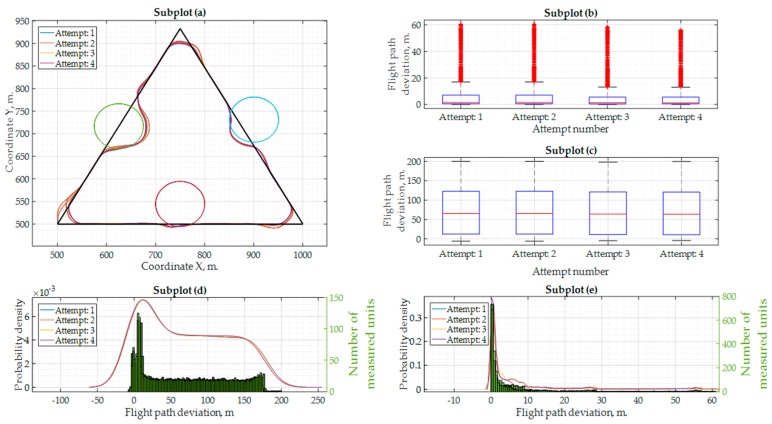
Results of the Software-in-the-Loop simulation with the “ArduPilot” autopilot with the flight speed at 10 m/s: (**a**) flight mission, flight trajectory and ground obstacles; (**b**) flight deviation from the standard flight mission path (when using ground obstacle avoidance); (**c**) flight deviation from the ground obstacle area boundary of 50 m. (when using ground obstacle avoidance); (**d**) probability density values and quantity of measurements for every flight path deviation value (standard mission); (**e**) probability density values and quantity of measurements for every flight path deviation value (from ground obstacle area boundary).

In Table 5, the minimum and maximum values of the flight path deviation are provided together with a mean µ and standard deviation σ for flight accuracy. The first values in the Table 5 represent the statistics obtained when using our new algorithm (distance is measured from 50 m. boundary area circles) and the values in brackets represent the statistics obtained when the UAV’s position is measured from the standard flight mission path. It was realized that the UAV delay would be smaller if the flight speed was reduced. Therefore, the same Software-in-the-Loop experiment was repeated for the second time, with a 10 m/s flight speed of the UAV. The results for this experiment are shown in Figure 11. It is evident from Figure 11a that the UAV flight was more accurate and the GNSS position values were closer to the theoretical calculations of the path coordinates. In Figure 11b,c, the first and even second quartiles were closer to 0 m, which is a good sign for the required flight accuracy. The probability density functions remained approximately the same as for the previous experiment. More Software-in-the-Loop simulation results for a flight speed at 10 m/s are shown in Table 6.

**Figure 12:**
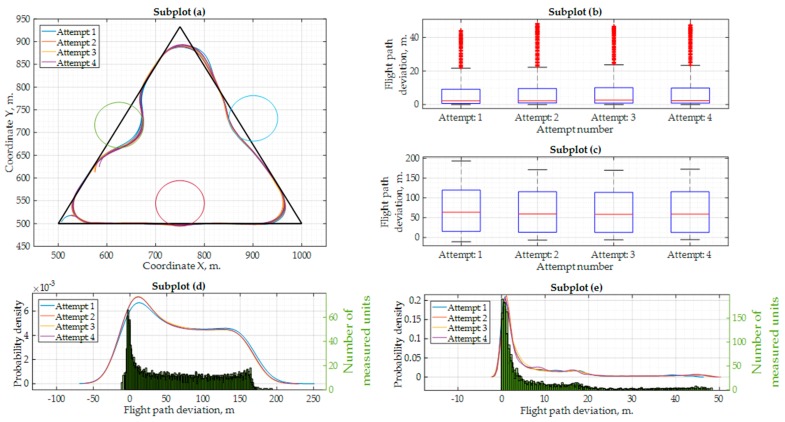
Results of real flight experiments with the “PX4” autopilot with the flight speed at 15 m/s and using the “Ublox Neo-M8N” GNSS receiver: (**a**) flight mission, flight trajectory and ground obstacles; (**b**) flight deviation from the standard flight mission path (when using ground obstacle avoidance); (**c**) flight deviation from the ground obstacle area boundary of 50 m. (when using ground obstacle avoidance); (**d**) probability density values and quantity of measurements for every flight path deviation value (standard mission); (**e**) probability density values and quantity of measurements for every flight path deviation value (from ground obstacle area boundary).

Flight experiments were carried out with a “PX4” autopilot. This autopilot was chosen because of a very short delay time for decision-making. This flight experiment was done with a UAV flying at 15 m/s and experiencing a 2–3 m/s southwest wind. The results are shown in Figure 12 and more data is presented in Table 7.

In this scenario, as seen in Figure 12a, the UAV flies around the imaginable ground obstacles much more accurately. The UAV makes instant decisions, and the flight trajectory comes much closer to the theoretically calculated one. For this flight experiment, all four attempts were done with the same flight configuration, since no changes can be made for UAV delay tuning with this autopilot. Some overshoot is still seen near the coordinate WP_2_. It is obvious that it was caused by the southwest wind.

**Figure 13:**
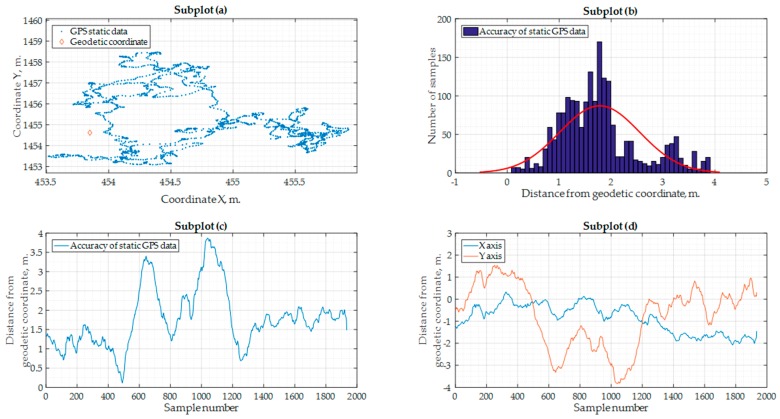
Accuracy of the “Ublox Neo-M8N” GNSS receiver when the data is measured by using a precise geodetic coordinate: (**a**) GNSS receiver measurement drift from the accurately measured geodetic coordinate; (**b**) Gaussian distribution of GNSS samples; (**c**) GNSS receiver measurement error; (**d**) GNSS receiver measurement error in X and Y directions.

Although the accuracy of the GNSS receiver which was used for the coordinate measurements is unknown, an assessment of the received coordinate accuracy was carried out. The GNSS “Ublox Neo-M8N” was put stationary on the geodetic coordinate 54.67192105° N 25.506942669° E. The static reading of this coordinate was measured, and, for comparison, the coordinates were transformed from the geodetic into the ENU frame. The GNSS data results are illustrated in Figure 13. As seen in Figure 13b,c, the common static data drifted from the correct position coordinate up to 4 m. Also, as seen from Figure 13d, the latitude uncertainty varied up to 4 m, whereas the longitude uncertainty was up to 2 m. These values could be subtracted or added to the data provided in Table 2, Table 3 and Table 4. For example, if we take the GNSS error into consideration and subtract this value from the data in Table 4, where real flight experiment data is provided, it is possible that our new algorithm could be applied for the safe avoidance of ground obstacles.

## 7. Conclusions

The results of the experiments showed that the “ArduPilot” autopilot is unable to fly around the obstacles as accurately as the theoretical calculations predict. This problem arises due to this autopilot’s decision lag. Due to this lag, the UAV makes some overshoots and undershoots near the turn or in cases when a flight manoeuver has to be made around the obstacle. Since our theoretical calculations only evaluate the initial turn lag when an obstacle is detected, the calculations could be improved by recognizing that this decision delay will appear for all additional waypoints that are required to fly around the obstacle safely. Also, in order to have smaller overshoots or undershoots, it might be possible to find an optimal “ArduPilot” autopilot flight configuration for different turns.

Due to this decision delay of the “ArduPilot” autopilot, for real flight experiments the “PX4” autopilot was chosen due to a greater processing power and a lower decision lag. The flight tests showed that the coordinate results for the flight path were much closer to the theoretically calculated trajectory. The results proved that the decision delay is an important factor for ground obstacle avoidance. Although the 50 m boundary area around the ground obstacle was still crossed and the maximum distance by which the safety rule was violated increased from an average of −6.09 m (Software-in-the-Loop) to −6.52 m (“PX4”). However, the average flight path error, maximum flight path error and standard deviation of the flight path error have decreased.

Real flight experiment results were influenced by the 2–3 m/s variable southwest wind, due to which some overshoots are seen near the second mission waypoint. Also, as a low cost GNSS is used to collect the coordinate data, a flight path error of up to 4 m may be caused by the GNSS data inaccuracy. Nevertheless, in general, the new algorithm works as expected if we would consider that there was a negative influence of the GNSS inaccuracy.

The new algorithm for ground obstacle avoidance can be implemented as an autopilot software for more advanced testing. In further research, more attention should be devoted to wind impact on safe ground obstacle avoidance, the influence of the autopilot’s decision delay on flight accuracy and GNSS data adaptation to reduce the influence of the GNSS on flight safety when a UAV is flying around ground obstacles.

## Figures and Tables

**Table 1:** Main technical characteristics for GNSS receiver “Ublox Neo-M8N”.

Ublox Neo-M8N	
Satellite systems	GPS/QZSS; GLONASS; Galileo; BeiDou
Supply	2.7–3.6 V; Lowest power (DC/DC)
Interfaces	UART; USB; SPI; I^2^C
Features	Flash; Data logging; Additional SAW and LNA; RTC crystal; Active antenna

**Table 2:** “Ublox Neo-M8N” accuracy and limits for various operational modes.

Accuracy or Type of Limit	Mode	GPS/GLONASS	GPS/BeiDou	GPS
Time to first fix	Cold start	26 s	27 s	29 s
	Hot start	1 s	1 s	1 s
	Aided starts	2 s	3 s	2 s
Sensitivity	Navigation	−164 dBm	−162 dBm	−163 dBm
	Reacquisition	−159 dBm	−159 dBm	−159 dBm
	Cold start	−147 dBm	−147 dBm	−147 dBm
	Hot start	−156 dBm	−156 dBm	−156 dBm
Max nav update rate		5 Hz	5 Hz	10 Hz
Velocity accuracy		0.05 m/s	0.05 m/s	0.05 m/s
Heading accuracy		0.3°	0.3°	0.3°
Horizontal accuracy	Autonomous	2.5 m	2.5 m	2.5 m
	SBAS	2.0 m	2.0 m	2.0 m
Operational limits	Dynamics	≤4 g	≤4 g	≤4 g
	Altitude	50,000 m	50,000 m	50,000 m
	Velocity	500 m/s	500 m/s	500 m/s

**Table 3:** “Pixhawk 2” autopilot technical specification.

Pixhawk 2	
Processor	32bit STM32F427 Cortex M4 core with FPU; 168 MHz; 256 KB RAM; 2 MB Flash; 32 bit STM32F103 failsafe co-processor;
Sensors	ST Micro L3GD20H 16 bit gyroscope; ST Micro LSM303D 14 bit accelerometer/magnetometer; Invensense MPU 6000 3-axis accelerometer/gyroscope; MEAS MS5611 barometer

**Table 4:** ENU coordinates of the mission points and ground obstacles.

	WP_1_	WP_2_	WP_3_	O_1_	O_2_	O_3_
E (m)	500	750	1000	625	900.51	750
N (m)	500	933.01	500	716.51	731.24	544.32
U (m)	50	50	50	50	50	50

**Table 5:** Results of the flight path deviation for the SITL experiment with a 15 m/s UAV speed.

	Min (m)	Max (m)	μ (m)	σ (m)
Attempt 1	−13.29 (0.01)	197.34 (67.39)	79.20 (11.01)	62.27 (15.00)
Attempt 2	−11.03 (0.02)	197.99 (66.63)	77.07 (10.69)	60.78 (14.68)
Attempt 3	−6.99 (0.00)	198.93 (62.41)	74.61 (9.31)	59.45 (13.68)
Attempt 4	−5.93 (0.01)	197.77 (58.89)	72.72 (8.29)	58.50 (12.94)

**Table 6:** Results of the flight path deviation for the SITL experiment with a 10 m/s UAV speed.

	Min (m)	Max (m)	μ (m)	σ (m)
Attempt 1	−8.81 (0.00)	197.61 (63.66)	73.37 (8.22)	59.04 (13.17)
Attempt 2	−6.06 (0.00)	199.69 (60.70)	71.99 (7.25)	58.03 (12.77)
Attempt 3	−5.02 (0.00)	197.94 (58.51)	70.71 (6.81)	57.39 (12.51)
Attempt 4	−4.49 (0.00)	199.26 (56.31)	70.52 (6.69)	57.27 (12.45)

**Table 7:** Results of the flight path deviation for real flight experiments with a UAV speed of 15 m/s and a 2–3 m/s southwest wind.

	Min (m)	Max (m)	μ (m)	σ (m)
Attempt 1	−10.93 (0.00)	193.27 (44.22)	69.54 (6.67)	55.98 (9.49)
Attempt 2	−6.79 (0.00)	171.22 (48.32)	66.59 (7.43)	54.95 (10.83)
Attempt 3	−5.22 (0.01)	170.00 (46.64)	65.81 (7.59)	55.59 (10.84)
Attempt 4	−3.14 (0.01)	172.56 (47.52)	66.29 (7.54)	55.03 (10.86)
